# Comparison of Classical and Inverse Calibration Equations in Chemical Analysis

**DOI:** 10.3390/s24217038

**Published:** 2024-10-31

**Authors:** Hsuan-Yu Chen, Chiachung Chen

**Affiliations:** 1 Africa Industrial Research Center, National Chung Hsing University, Taichung 40227, Taiwan; wakaharu37@gmail.com; 2 Department of Bio-Industrial Mechatronics Engineering, National Chung Hsing University, Taichung 40227, Taiwan

**Keywords:** calibration, classical equation, inverse equation, predictive performance

## Abstract

Chemical analysis adopts a calibration curve to establish the relationship between the measuring technique’s response and the target analyte’s standard concentration. The calibration equation is established using regression analysis to verify the response of a chemical instrument to the known properties of materials that served as standard values. An adequate calibration equation ensures the performance of these instruments. There are two kinds of calibration equations: classical equations and inverse equations. For the classical equation, the standard values are independent, and the instrument’s response is dependent. The inverse equation is the opposite: the instrument’s response is the independent value. For the new response value, the calculation of the new measurement by the classical equation must be transformed into a complex form to calculate the measurement values. However, the measurement values of the inverse equation could be computed directly. Different forms of calibration equations besides the linear equation could be used for the inverse calibration equation. This study used measurement data sets from two kinds of humidity sensors and nine data sets from the literature to evaluate the predictive performance of two calibration equations. Four criteria were proposed to evaluate the predictive ability of two calibration equations. The study found that the inverse calibration equation could be an effective tool for complex calibration equations in chemical analysis. The precision of the instrument’s response is essential to ensure predictive performance. The inverse calibration equation could be embedded into the measurement device, and then intelligent instruments could be enhanced.

## 1. Introduction

Calibration is essential to ensuring the performance of sensors or instruments in chemical analysis. Many quantitative analytical techniques, such as high-performance liquid chromatography (HPLC), ultraviolet–visible spectroscopy (UV-Vis spectroscopy), gas chromatography/mass spectrometry (GC-MS), and electrophoresis, must establish a calibration equation to express the relationship between the measuring technique’s response and the target analyzer’s standard values [1,2].

In practical work, standard concentrations or environments are prepared. These values are called regressors or independent values, x. The responses from the measuring technique are called the dependent values, y. The data sets (x_i_, y_i_) model the relationship between x and y. The practical measuring case detects a sample with unknown conventions, x_0_; the new response is y_0_. The new concentration, x_0_, will be calculated using the previously established calibration equation [3,4].

Two types of calibration equations were proposed. The first calibration equation is the classical calibration. The function of this equation is *y_i_* = *f*(*x_i_*). If the dependent variable y_i_ and the independent variance, x_i_, exist in the linear relationship, the calibration model is [5]

(1) $$y_{i}=b_{0}+b_{1}x_{i}+\varepsilon_{i}$$

where $b_{0}$ is the intercept, $b_{1}$ is the slope, and $\varepsilon_{i}$ represents random errors.

The assumptions of regression analysis are that the $\varepsilon_{i}$ represents random errors, independent, and normal distribution, and the *x_i_* values are non-measurement errors [6].

In a further application, the unknown *x*_0_ was detected. The y_0_ was detected by measuring instrument, and the predicted ${\hat{x}}_{0}$ value is calculated as

(2) $$\;{\hat{x}}_{0}=\frac{y_{0}-b_{0}}{b_{1}}$$

The second calibration is called the inverse equation. The function of this equation is x_i_ = g(y_i_). In the inverse regression, x_i_ is treated as the response, and y_i_ is the regressor. If the linear relationship exists, the calibration model is

(3) $$x_{i}=c_{0}+c_{1}y_{i}+\varepsilon_{i}$$

As the new response, x_0_, is detected, the predicted ${\hat{x}}_{0}$ value is calculated directly using Equation (3).

(4) $${\hat{x}}_{0}=c_{0}+c_{1}y_{0}$$

One of the regression analysis assumptions is the negligible measurement error for the x_i_ value. For the inverse equation, the assumption is invalid. However, different opinions are presented in different studies. Krutchkoff [7] compared the classical and inverse equations using the Monte Carlo methods and found the inverse equation had a lower mean square error value. Krutchkoff [8] found the inverse equation demonstrates better extrapolation performance. Centner [9] compared two calibration equations using Monte Carlo methods and two practical examples and found the inverse equation to be more reliable than the classical one. Tellinghuisen [10] evaluated two calibration equations for small data sets and claimed that the inverse equation is more efficient over an extensive range of the variable x_i_. Shalabh [11] compared the measurement errors of two calibration equations with the balanced loss function. He suggested that the comparison should not be limited to linear equations and that more variables and nonlinear relationships should be considered.

Tellinghuisen [12] proposed sample algorithms for nonlinear calibration equations of the classical equation to calculate the prediction of new measurement values easily. However, this study did not mention the convenience of the inverse equation. Parker et al. [13] claimed that the inverse linear equation is simple and easy to use but violates some regression assumptions and found the inverse linear equation had more variability and bias in the prediction interval as the predicted value is away from the center of the data. In the study of Besalu [14], the inverse calibration has a better prediction ability than the classical calibration equation and gives lesser mean square error interpolations. Granovskii and Sirala [15] presented different conditions and included a known parameter to estimate experimental data and homo-variance when selecting classical or inverse calibration equations. Witkovsky and Wimmer [16] introduced a method to calculate the measurement uncertainty of polynomial equations for two calibration equations. Delgado [17] used the Beer–Lambert law to illustrate the misuse of the calibration equations for this calibration curve. To predict new measurements, the hypothesis of regressions does not necessarily have to be fulfilled for the nonlinear equation, and the inverse equation has a more extraordinary predictive ability than the classical equation.

Francois et al. [18] proposed two criteria to evaluate calibration equations: the maximum and the average prediction variance, and concluded that the predictive quality of both calibration equations was equal. Kannan et al. [19] compared the classical and inverse equations with the Pitman closeness criterion and showed that the inverse equation had better predictive ability than the classical equation as the calibration point is far from the average values of standards.

Most of the literature concerns only the linear relationship of the calibration equation. Delgado [17] mentions the misuse of linear equations in calibration equations. Chen and Chen [20] proposed six calibration equations for calibration curves in chemical analysis. The linear equation is one of their calibration equations. In this study, the calibration curves of two types of hygrometers were established. These data were divided into two sets: one for the model established and the other for evaluating the accuracy and precision of the adequate equation. Nine data sets were collected from previous studies to evaluate the predictive performance of two calibration equations. These data sets are listed in Table 1. Four criteria were proposed to evaluate the predictive ability of two calibration equations. The effect of the instrument’s precision on the predictive ability was assessed.

## 2. Materials and Methods

### 2.1. Relative Humidity Sensors

This study used two types of humidity sensors: a capacitive Vaisala HMP-143A (Vaisala Oyj, Vantaa, Finland) and a resistive Shinyei THI-B141 (Shinyei Kaisha Technology, Kobe, Japan). The specifications of these sensors are listed in Table 2.

### 2.2. Saturated Salt Solutions

Eleven saturated salt solutions, made from LiCl, CH_3_COOK, MgCl_2_, K_2_CO_3_, Mg(NO_3_)_2_, NaBr, KI, NaCI, KCI, KNO_3_, and K_2_SO_4_, were used to maintain the standard relative humidity values for calibration. The standard humidity values produced by these salt solutions were listed using Greenspan’s equation [30].

### 2.3. Calibration of Humidity Sensors

Two types of humidity sensors were calibrated using saturated salt solutions. The procedures for producing saturated salt solutions were according to the OIMO R121 [31]. Chen’s study [32] describes the detailed calibration procedures. Both data sets, the response data of sensors, and the standard values from saturated salt solutions for two types of humidity sensors were used to compare the classical and inverse calibration equations.

### 2.4. Establish the Calibration Equation

The standard humidity values of a saturated salt solution are called regressors or independent variables, x_i_. The reading values of humidity sensors are called response or dependent variables, y_i_.

#### 2.4.1. The Classical Equation

The form of this equation is a high-order polynomial equation.

y = b_0_ + b_1_x + b_2_x^2^+ … + b_k_x^k^ (5)

where b_0_, b_1_, b_2_, and b_k_ are constants.

#### 2.4.2. The Inverse Equation

The form of the inverse equation is

(6) $$x=c_{0}+c_{1}y+c_{2}y^{2}+\cdots ..c_{n}y^{n}$$

where *c*_0_, *c*_1_, *c*_2_, and *c_n_* are constants.

### 2.5. The Evaluation Criteria for Calibration

The Criteria of Fitting Agreement

The quantitative criteria for assessing the fitting ability for different calibration equations of the same data sets are the coefficients of determination, R^2^, and the standard error in the estimate errors, s. The qualitative criterion is the residual plot [6,33,34].

For the classical equation, the standard error in the estimate errors, *s_x_*, is

(7) $$s_{x}=\frac{\sqrt{\Sigma {\left(y_{i}-{\hat{y}}_{i}\right)}^{2}}}{n-p}$$

where $y_{i}$ is the dependent variable, $\hat{yi}$ is the predated value for this calibration equation, *n* is the number of data points, and *p* is the number of parameters.

For the inverse equation, the standard error in the estimate errors, *s_y_*, is

(8) $$s_{y}=\frac{\sqrt{\Sigma {\left(x_{i}-{\hat{x}}_{i}\right)}^{2}}}{n-p}$$

where $x_{i}$ is the dependent variable, $\hat{xi}$ is the predated value for this calibration equation, n is the number of data points, and p is the number of parameters.

The residual plots are used to assess the fitting ability of these equations. If the residual plots’ distribution was uniform, this equation was adequate. If a fixed pattern was found for the residual plots, it showed that this equation was inadequate. More variables need to be considered.

### 2.6. Compare the Predictive Performance for Two Calibration Equations

#### 2.6.1. The Criteria for the Predictive Performance of Two Calibration Equations

Four criteria were proposed for the comparison of predictive performance [35].

The predictive errors were defined as the difference between predicted values from calibration equations and standard values:

(9) $$e_{i}=x_{i0}-{\hat{x}}_{i0}$$

where x_i_ is the standard value, and ${\hat{x}}_{i0}\;$ is the measurement value calculated by calibration equations.

1. The minimum $e_{i}$ value, e_i,min_.
2. The maximum $e_{i}$ value, e_i,max_.
3. Mean absolute error (MAE):
  (10) MAE=Σein
  where $\left|e_{i}\right|$ is the absolute $e_{i}$ value.

MAE is used to evaluate the accuracy of the equation. The smaller the MAE, the greater the accuracy of the predicted equation.

4. Root mean square error (RMSE):
  (11) $$RMSE={\left(\frac{\Sigma {e_{i}}^{2}}{n}\right)}^{0.5}$$

RMSE is used to evaluate the precision of the equation. The smaller the RMSE, the greater the precision of the predicted equation.

#### 2.6.2. The Criteria for the Comparison of the Predictive Performance of Two Calibration Equations

Two criteria were proposed to compare the predictive performance of the two calibration equations for the same data sets:

RE_MAE_ = (MAE_cla_ − MAE_inv_)/MAE_cla_ (12)

where MAE_cla_ is the MAE value of the classical equation, and MAE_inv_ is the MAE value of the inverse equation.

RE_RMSE_ = (RMSE_cla_ − RMSE_inv_)/RMSE_cla_ (13)

where RMSE_cla_ is the RMSE value of the classical equation, and the RMSE_inv_ is the RMSE value of the inverse equation.

The RE_MAE_ and RE_RMSE_ compare the accuracy and precision of two calibration equations. If MAE_cla_ > MAE_inv_, the inverse equation has better accuracy, and the RE_MAE_ value is positive. The RE_MAE_ values also showed the degree of the two calibration equations’ different accuracy abilities.

If RMSE_cla_ > RMSE_inv_, the inverse equation has better precision, and the RE_RMSE_ value is positive. The RE_RMSE_ values also showed the degree of the difference in precision between the two calibration equations.

### 2.7. Data Splitting

All data measured from two humidity sensors or collected from the literature were divided into two data sets. The first data set was used to evaluate the adequate form of the calibration equation, and the second was used to evaluate the equation’s predictive ability.

For example, sixty-six values (x_i_, y_i_) were collected from a humidity sensor. The thirty-three values from (x_1_, y_1_) to (x_33_, y_33_) were used to assess the adequate form of the classical equation y_i_ = f(x_i_) and the inverse equation x_i_ = g(y_i_). The response value, y_i_, of the other 33 data points (y_34_ to y_66_) were substituted into f(x_i_) and g(y_i_) equations to calculate the predicted values (${\hat{x}}_{34}\;to\;{\hat{x}}_{66})$. The difference between x_i_ (standard values) and ${\hat{x}}_{i}$ (calculated values) is the predicted error, *e_i_*.

### 2.8. The Calculation of the New Measurement

After the calibration equations are established, the new observation from the sensors is *y*_0_. The calculation of the new measurement values, *x*_0_, is different for two calibration equations.

1. The inverse equation.

The new measurement value x_0_ is calculated directly from this equation.

(14) $$\;\;\;\;{\hat{x}}_{0}=c_{0}+c_{1}y_{0}+c_{2}{y_{0}}^{2}+\ldots +c_{n}{y_{0}}^{2}$$

2. The classical equation.

For the linear equation, $y_{0}=b_{0}+b_{1}x_{0}$, and ${\hat{x}}_{0}$ is calculated by

(15) $${\hat{x}}_{0}=(y_{0}-b_{0})/b_{1}$$

For the two other polynomial equations, $y_{0}=b_{0}+b_{1}x_{0}+b_{2}{x_{0}}^{2}$, and ${\hat{x}}_{0}$ is calculated by

(16) $${\hat{x}}_{0}=\frac{-b_{1}\pm {\left({b_{1}}^{2}-4(b_{2}\left(b_{0-}y_{0}\right))\right)}^{0.5}}{{2b}_{2}}$$

For high-order polynomial equations, *y*_0_ = *b*_0_ + *b*_1_*x* +$b_{2}{x_{0}}^{2}\;$+… + *b*_*k*_*x*^*k*^; the calculation of *x*_0_ is very complex and solved by numeric software (The Jenkins-Traub UDF).

### 2.9. Data Source for Comparing Two Calibration Equations

Nine data sets from the literature were collected to compare the predictive ability between the classical and inverse equations. The published literature is shown in Table 2. All original data for the response of chemical analysis instrumentation was divided into two data sets: one for the model established and the other for evaluating the accuracy and precision of the adequate calibration equation.

Adequate classical and inverse equations were established with modeling data sets. Then, the evaluation data of the response were substituted into these equations to calculate the measurement value. The predicted errors were used to compare the performance.

The forms of the calibration equations for the literature data are as follows [20]:

1. Higher-order polynomial equation:

y = b_0_ + b_1_x + b_2_x^2^ + … + b_k_x^k^ (17)

2. Exponential decay equation:

y = d_0_*exp(−d_1_*x) (18)

3. Power equation:

y = e_1_*x*^e2^ (19)

4. Exponential rise to maximum equations (ERTM equations):

y = f_1_ (1 − Exp(−f_2_*x*)) (20)

## 3. Results

### 3.1. The Capacitive Humidity Sensor

#### 3.1.1. The Calibration Equation of Capacitive Humidity Sensors

**Figure 1:**
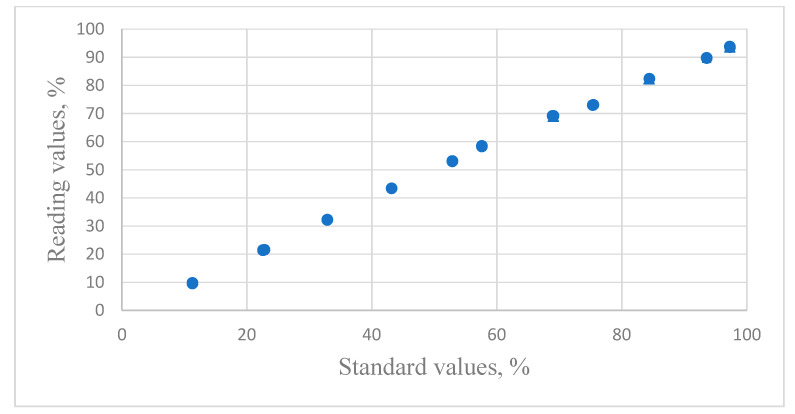
The distribution of the relative humidity data for reading values versus the standard humidity values for Vaisala HMP-143A capacitive sensors.

Figure 1 presents the relationship between the reading values of capacitive humidity sensors and the standard values produced by the saturated salt solutions.

1. The classical equation

From the criteria, the adequate calibration equation established from the calibration data of the capacitive sensor is

y = −3.60919 + 1.1626x − 0.0017933 x^2^ (21)

where R^2^ = 0.9996, and s_y_ = 0.5780.

For the new response y_0_, the corresponding values of the new measurement are calculated as follows:

(22) $$y_{0}=-3.60919+1.1626x_{0}-0.0017933{x_{0}}^{2}$$

(23) $$x_{0}=338.0968\pm 290.811{\left(1.326816-6.87731\times 10^{-3}y_{0}\right)}^{0.5}$$

2. The inverse equation of capacitive humidity sensors

From the criteria, the adequate calibration equation established from the calibration data of the capacitive sensor is

x = 3.5299 + 0.83231y + 0.001842y^2^ (24)

where R^2^ = 0.995, and s_x_ = 0.6344.

For the new response of $y_{0}$ from sensors, the corresponding value of the new measurement is calculated directly.

(25) $$x_{0}=3.5299+0.83231y_{0}+0.001842{y_{0}}^{2}$$

#### 3.1.2. The Evaluation of the Calibration Equation of Capacitive Humidity Sensors

The other data set, independent of the previous data sets used to establish the calibration equation, is used to evaluate the predictive performance of two types of calibration equations. The new data sets (x_1_′, y_1_′), (x_2_′, y_2_′), … (x_n_′, y_n_′) were substituted into Equations (23) and (25).

The difference between the standard and calculated measurement values is e_iy_ and e_ix_. Table 3 lists four criteria: e_i_,_min_, e_i_,_max_, MAE, and RMSE.

In Table 3, the inverse equation has a smaller e_i,min_ value and a larger e_i,max_ value. The inverse equation’s MAE and RMSE values are smaller than those of the classical equation. The MAE is the accuracy of the calibration equation, and the RMSE is its precision. The inverse calibration equation has better predictive performance than the classical equation for capacitive humidity sensors.

### 3.2. The Resistive Humidity Sensor

**Figure 2:**
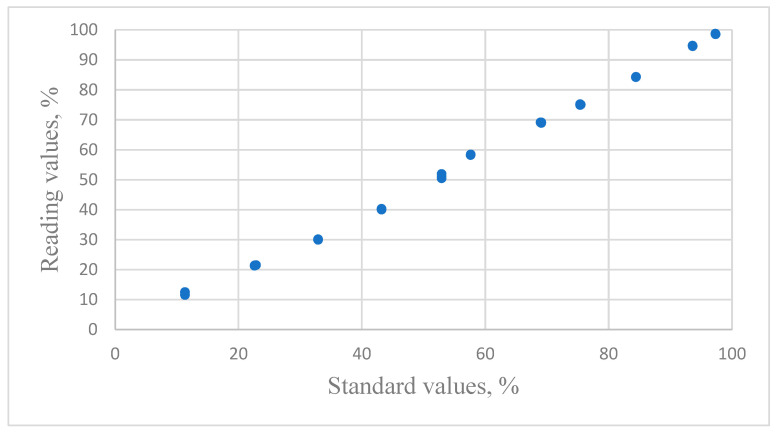
The distribution of the relative humidity data for reading values versus the standard humidity values for THT-B121 resistive sensors.

The relationship between the reading values of resistive humidity sensors and the standard values is shown in Figure 2.

#### 3.2.1. The Calibration Equation of Resistive Humidity Sensors

The adequate calibrations established from the calibration data of the resistive sensor established by regression analysis are described below:

1. The classical equation.

(26) $$y=5.8524+0.4728x+0.0099x^{2}-5.1025\times 10^{-5}x^{3}$$

where R^2^ = 0.996, and s_y_ = 0.5757.

For the new response of this humidity sensor, y_0_, the measurement value, $x_{0}$, is calculated by the following equation:

(27) $$5.1025\times 10^{-5}{x_{0}}^{3}-0.0099{x_{0}}^{2}-0.4729x_{0}-5.8524+y_{0}=0$$

Equation (27) is a three-order polynomial equation; the *x*_0_ value needs to be solved by numeric software.

2. The inverse equation.

The adequate calibration equation established from the calibration data of the capacitive sensor is

(28) $$x=-6.1370+1.5446y-0.0103y^{2}+5.0\times 10^{-5}\;y^{3}$$

where R^2^ = 0.996, and s_x_ = 0.6344.

For the new response, y_0_, the measurement, x_0_, can be calculated directly.

(29) $$x_{0}=-6.1370+1.5464y_{0}-0.0103{y_{0}}^{2}+5.3\times 10^{-5}{y_{0}}^{3}$$

#### 3.2.2. The Evaluation of the Calibration Equation of Resistive Humidity Sensors

The other data set, independent of the data sets used to establish calibration equations, was used to evaluate the predictive ability.

The criteria for the evaluation of two equations are listed in Table 4.

The classical equation has smaller e_i,min_, e_i,max_, and MAE values. The inverse calibration equation has a smaller RMSE value. However, the differences in these criteria between the two calibration equations were limited. No significant difference could be found in the predictive performance of the two equations. However, the new measurement values of the inverse equation can be calculated directly. The calculation of new measurement values for the classical equation is very complex.

### 3.3. The Evaluation of Two Calibration Equations from Previous Data in the Literature

#### 3.3.1. The Measurement of Chloromethane Concentration with GC-MS

**Figure 3:**
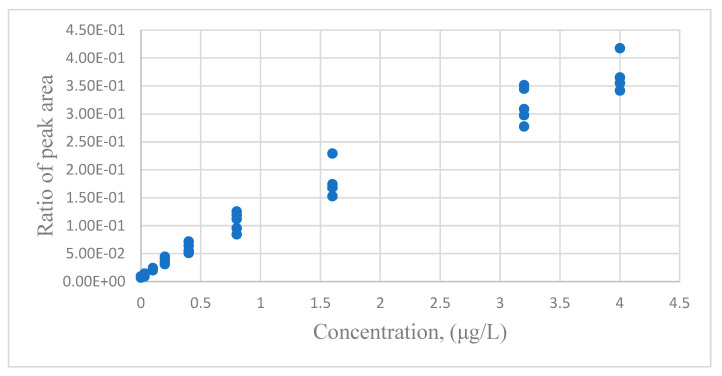
The distribution of the chloromethane data for the ratio of peak areas versus the standard concentrations for GC-MS.

Lavagnini and Magno [23] measured chloromethane concentration with GC-MC. There are nine concentration levels (μg/L) and ten replicates for each concentration. The first data set contained 45 data points for establishing the calibration equation. The other 45 data points were used to evaluate the predictive ability. The distribution between the response of the peak area and the standard concentration is shown in Figure 3. The regression analysis results of two calibration equations are listed in Table 5.

From the criteria for different equations, the adequate calibration equation for the classical equations is

(30)  y=0.0109+0.7477(1−exp⁡(−0.1585x))

For the new response (the ratio of peak area), y_0_, the measurements of two equations are calculated as follows:

(31) $$x_{0}=-6.30915Ln(1.01458-1.3374\;y_{0})$$

The criteria for evaluating these calibration equations are listed in Table 6.

The results indicated that the inverse equation had smaller values for these criteria than the classical equation.

#### 3.3.2. Using Spectrophotometry to Measure Albumin

**Figure 4:**
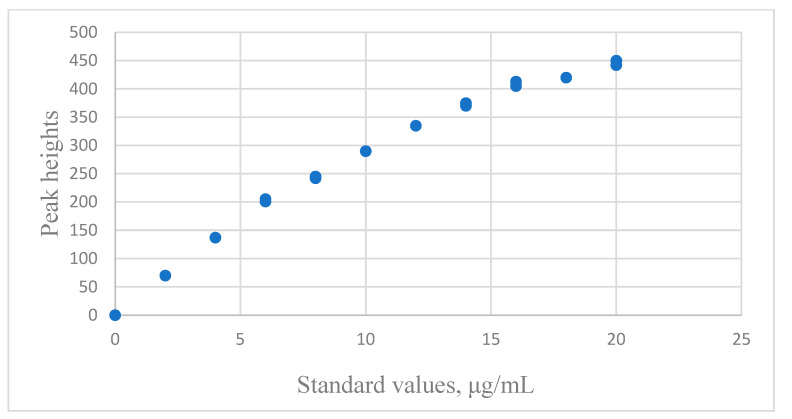
The distribution of the albumin concentration data for the peak heights versus the standard concentrations with spectrophotometry.

In the test conducted by Rawski et al. [25], there were 11 albumin standards (μg/mL) and three replicates for each concentration. The response of this instrument, y_i_, was the peak height. The distribution between response and standard values of model development data is shown in Figure 4. The results of the regression analysis are listed in Table 7.

The adequate calibrations are listed as follows:

1. The classical equation is

(32) y=626.5993(1−exp⁡(−0.0635x))

For a new response, y_0_, the new measurement of x_0_ could be calculated by

x_0_ = −15.748 Ln(1 − y_0_/626.5933) (33)

2. The inverse equation is

(34) $$x=0.2443+0.0164_{y}+5.8629\times 10^{-5}y^{2}$$

The new measurement, $x_{0},$ can be calculated directly using an equation.

The criteria for evaluating predictive ability for two calibration equations are listed in Table 8.

The classical equation had better predictive ability. The MAE and RMSE values showed better accuracy and precision in the classical equation.

#### 3.3.3. The Measurement of Anti-IgG by Biophotonic Sensing Cells

The calibration of a biochip composed of a set of BICELLS (biophotonic sensing cells) for anti-IgG [29] was studied for adequate calibration equations. The response (y_i_) is the transduction signal, and the standard concentration (μg/mL) is the regressor (x_i_). The calculation regression results are as follows:

The classical equation is

(35) $$y=-0.3135+2.109x-0.002827x^{2}+1.335\times 10^{-5}x^{3}$$

where R^2^ = 0.9863, and s_y_ = 0.2331.

The inverse equation is

(36) $$x=-0.2732+12.8733y-5.259y^{2}+1.0162y^{3}$$

where R^2^ = 0.9833, and s_x_ = 0.1957.

The comparison of the predictive performance of two calibration equations is listed in Table 9.

The classical equation performed better in terms of e_i,min_, e_i,max_, and RMSE (precision), and two calibration equations had similar predictive performance for the MAE values.

#### 3.3.4. The Measurement of Drug Concentration in Blood with an HPLC Assay

The drug concentration in blood ($x_{i}$) was measured with an HPLC assay, and the response ($y_{i}$) ranged from 0.002 to 0.272 [27].

The adequate calibration equations are as follows:

1. The classical equation is
  (37) $$y=0.0199+1.3940\left(\;1-\exp\left(-0.5084x\right)\right)$$
  where R^2^ = 0.9921, and s_y_ = 0.0348.
2. The inverse equation is
  (38) $$x=-2.7006+3.7037y^{0.5377\;}$$
  where R^2^ = 0.9912, and s_x_ = 0.0896.

The predictive performance of the two calibration equations is listed in Table 10.

The class equation had smaller e_i,min_ values, and the inverse equation had smaller e_i,max_ values and better accuracy (smaller MAE) and precision (smaller RMSE). However, the predictive ability between two equations is not significantly different.

#### 3.3.5. Detection of EtP Compound by QqQ-MS

Martin et al. [28] reported determining EtP compound pollution by QqQ-MS. The instrument’s response is area ($y_{i}$), and the standard concentration is x_i_.

The adequate classical equation is

(39) $$y=-18360.5421+1577.4319x-0.1277x^{2}$$

where R^2^ = 0.9986, and s_y_ = 28310.3.

The adequate inverse equation is

(40) $$x=-0.5654+8.197\times 10^{-4}y-2.402\times 10^{-10}y^{2}+9.719\times 10^{-17}y^{3}$$

where R^2^ = 0.9998, and s_x_ = 28310.3.

The predictive performance of the two calibration equations is listed in Table 11.

The classical equation has lower values of e_i,min_, e_i,max_, and RMSE, which indicates better precision. The inverse equation has a lower MAE value.

#### 3.3.6. The Measurement of Sulfides by Flow Injection Analysis

Desimoni [22] reported the calibration data set of sulfides in flow injection analysis at a palladium-vitreous carbon-modified electrode. The x_i_ is the standard concentration, and the response y_i_ is the current (μA).

The adequate classical equation is

(41) $$y=0.1658x^{1.0369}$$

where R^2^ = 0.9998, and s_y_ = 0.117.

The adequate inverse equation is:

(42) $$x=596.3756\left(\;1-Exp\left(-0.009238y\right)\right)$$

where R^2^ = 0.9999, and s_x_ = 0.295.

The predictive performance of two calibration equations is listed in Table 12.

With smaller e_i,min_, e_i,max_, MAE, and RMSE values, the inverse equation performed better than the classical equation.

#### 3.3.7. Measurement of Daidzein by HPLC Analysis

Mulholland and Mibbert [21] detected the daidzein concentration with a HPLC analyzer. The standard concentration is x_i_ (mg/50 mL), and the HPLC response is the pear area (y_i_).

The adequate classical equation is

(43) $$y=-0.4955+3.222x^{0.9471}$$

where R^2^ = 0.9995, and s_y_ = 0.2459.

The adequate inverse equation is

(44) $$x=0.1535+0.2992y^{1.055}$$

where R^2^ = 0.9946, and s_y_ = 0.0845.

The predictive performance of the two calibration equations is listed in Table 13.

The classical equation has a lower e_i,min_ and RMSE (precision), and the inverse equation has a lower e_i,max_ and MAE (accuracy).

#### 3.3.8. Measurement of Cocaine Concentration by LC-MS-MS

Desharnais et al. [26] measured cocaine concentrations with an LC-MS-MS instrument. The standard concentration is x_i_, and the response, y_i_, is the instrument’s area ratio. The response data did not have a constant variance. These x_i_ values were logarithmic transformations which require further analysis.

The adequate classical equation is

y = 0.1832 + 0.008991 ln(x)^5.0619^ (45)

where R^2^ = 0.9994, and s_y_ = 0.1401.

The adequate inverse equation is

(46) $$Lnx=-30.5041+34.2735\;y^{0.3119}$$

where R^2^ = 0.9991, and s_x_ = 0.0604.

The predictive performance of the two calibration equations is listed in Table 14.

The classical equation has lower e_i_,_min_, and the inverse equation has lower e_i_,_max_, MAE (accuracy), and RMSE (precision).

#### 3.3.9. Measurement of Benzaldehyde Using Pulse Polarography

Ortiz et al. [24] reported the calibration data for determining benzaldehyde concentration by pulse polarography. The standard concentration (m/mol) is x_i_, and the current response (μA) is y_i_.

The adequate classical equation is

y = 2.0923 − 2.1131 Exp(−1.0328x) (47)

where R^2^ = 0.9762, and s_y_ = 0.0204.

The adequate inverse equation is

x = 0.0308 − 0.01089y +3.6389y^2^ − 6.0929y^3^ (48)

where R^2^ = 0.9821, and s_x_ = 0.0037.

The predictive performance of the two calibration equations is listed in Table 15.

The classical equation has lower e_i,max_, and the inverse equation has lower e_i,min_, MAE (accuracy), and RMSE (precision).

## 4. Discussion

The standard values (x_i_) are prepared without measurement errors for the calibration procedure. One of the regression assumptions is the negligible error for the xi value. For the classical equation, the x_i_ value of the standard values corresponded to this requirement. The instrument’s response, y_i_, is the regressor value. The measurement errors exist in the regressor variables. If the response variance is limited, the effect of measurement error on the regressor can be ignored with a minor effect [6].

The coefficient of variation (CV) can be used to evaluate the effect of measurement errors. The CV is defined as

CV = (s/y_mean_) × 100% (49)

where s is the standard deviation of the instrument’s responses and y_mean_ is the mean of the response values at the fixed standard value.

The results of comparing the predictive performance of two calibration equations for the measurement data of this study and the literature data are listed in Table 16. If the RE_MAE_ and RE_RMSE_ are positive, the inverse calibration equation has better accuracy and precision than the classical equation.

For the capacitive humidity sensor, the RE_MAE_ is 49.83%, and the RE_RMSE_ is 50.66%. This indicates that the inverse calibration equation has significantly better predictive performance than the classical calibration equation. The resistive humidity sensor’s RE_MAE_ is −1.93%, and the RE_RMSE_ is 1.78%. This shows that the classical equation has better accuracy, and the inverse equation has better precision. However, these differences in the predictive performance of resistive humidity sensors are insignificant.

**Figure 5:**
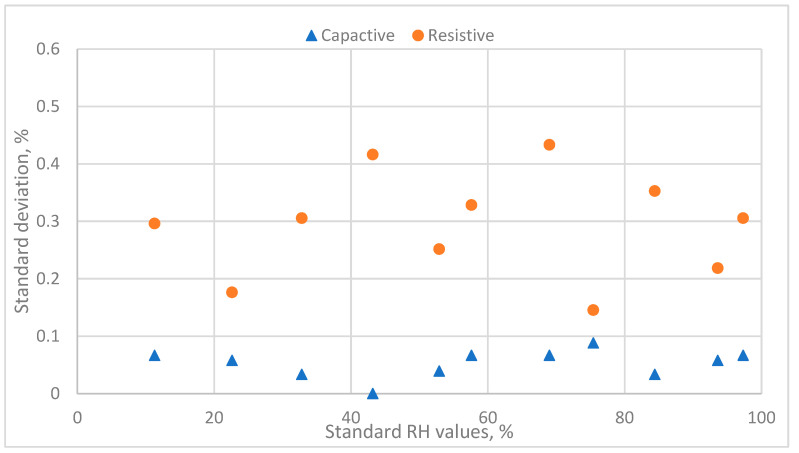
The distribution of the standard deviation values of the response and the standard relative humidity values for two humidity sensors.

Figure 5 indicates the data distribution of the standard deviation of the responses of two humidity sensors in the same standard humidity environment. The capacitive sensor has excellent replicative ability, with standard deviations < 0.1%. However, the resistive sensor’s standard deviations range from 0.14% to 0.44%. The larger standard deviations in the response in the same standard environment for resistive humidity sensors induced the problems. As the response (y_i_) is recognized as the dependent variable (regressor) and the measurement errors are significant, the severity of the assumption of regression analysis influences the predictive performance of the inverse calibration equation. In other words, smaller standard deviations of the measurement values could ensure the better predictive performance of the inverse calibration equation.

**Figure 6:**
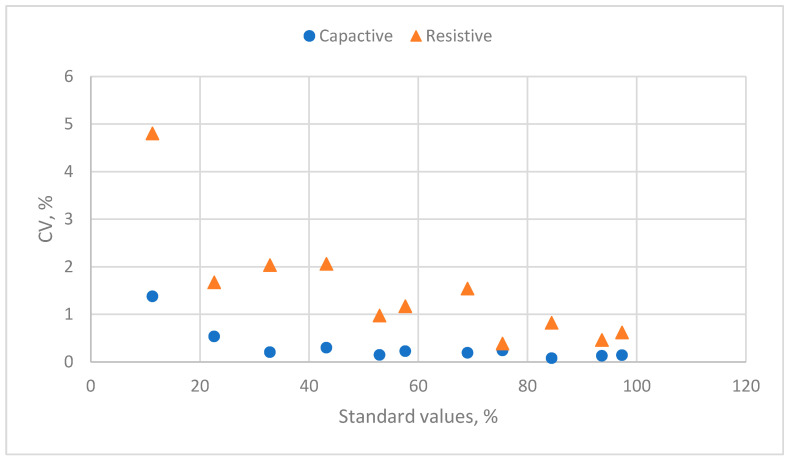
The distribution of the response’s CV values and the standard relative humidity values for two humidity sensors.

The distribution of the CV values of the response and standard humidity values is shown in Figure 6. The CV values of the capacitive humidity sensor were <0.6%. However, the resistive humidity sensor’s CV values range from 0.5 to 6.0%.

The inverse equation was first proposed last century. In this equation, the response values of instruments serve as independent variables, violating the assumption of no errors for the regressor. For this reason, many researchers adopted the classical calibration equation. However, calculating new measurement values from the new response of the instrument values is needed to transform the original classical equation. So, only the linear equation was selected as the adequate calibration equation. However, much of the literature indicates that high-order polynomial and nonlinear equations are adequate equations [11,13,17,20]. The transform forms of these classical calibration equations are very complex and impractical with regard to calculating the measurement values.

Francois et al. [18] concluded that two calibration equations have equal predictive ability. In this study, we found some calibration data sets have similar results, especially for the response values with a higher coefficient of variance (CV).

Many studies have mentioned the better-predicted performance of the inverse calibration equation [9,10,13,14]. Their results were based on small data sets or theoretical discussions. This study used the measurement data sets from two kinds of humidity sensors and nine data sets collected from the literature to evaluate the predictive performance of two calibration equations and confirmed the same results. Shalabh [11] mentioned that measurement errors must be considered for prediction accuracy. This study found the same results with the evaluation of the MAE values. Center [9] found an improvement in the predictive ability of inverse calibration with the decrease in measurement variance. In this study’s results, the inverse equation’s predicted predictive ability is superior to the classical equation if the CV values of the instrument’s response are smaller. Krutchkoff’s [7,8] study showed the inverse equation has a more minor average error. This study proposed the criterion of the RMSE values to confirm this statement.

In Table 16, the inverse calibration equation had significant predictive ability for GC-MS, flow injection analysis, LC-MS-MS, and pulse polarography. The CV values of the GC-MS, flow injection analysis, LC-MS-MS, and pulse polarography ranged from 4.0 to 8.2%, 0.3 to 1.7%, 0.4 to 1.4%, and 2.5 to 3.5%, respectively. The classical calibration equation performed similarly to the inverse calibration equation for spectrophotometry, BICELLS, HPLC, and QqQ-MS calibration data. The CV values of the spectrophotometry, BICELLS, HPLC, and QqQ-MS ranged from 3 to 24%, 13.5 to 24.5%, and 30 to 34%, respectively.

The results indicated that the better predictive performance of the inverse calibration equation is more significant than that of the classical equation if the response of this instrument has excellent replicative ability. The CV values of the response in each standard environment could serve as an index to express its replicative ability. In the case when the CV < 5%, the inverse calibration equation has a better predictive performance. In the case when the CV > 5%, the classical calibration equation has a better or similar predictive performance.

Recently, measurements of intelligent instruments have been used to embed calibration equations into this device. The inverse calibration equation can work well and easily. That is, an adequate inverse calibration equation could be used for intelligent measurement.

In the sensor industry, most of the sensors are physical sensors, accounting for 90%, while chemical sensors account for 9%, and biological sensors account for 1% [36]. Research on chemical sensors is, therefore, declining. However, chemical sensor application objects include medicine, food, the chemical industry, semiconductor manufacturing processes, etc. The measurement performance of chemical sensors has a significant impact. Chemical sensing requires the establishment of a calibration equation. Therefore, this paper should be valued in academic research.

Recently, researchers have been concerned about the impact of calibration equations on measurement performance. Their measurement objects include solvent-independent molecular weight [37,38], tiny mass [39], soil moisture [40], and pesticide concentrations [41]. The results of this study for two calibration equations could be applied to different instruments to improve their measurement performance.

## 5. Conclusions

There are two kinds of calibration equations: classical equations and inverse equations. The classical equation is widely used, and the linear equation is the main one used for calculating new measurement values. The inverse equation can compute the new measurement directly and efficiently. However, some researchers suspect it violates the basic assumptions of regression analysis. In this study, the actual calibration data sets of two types of humidity sensors were collected. Nine calibration data sets of various instruments were collected from the literature. Four criteria were proposed to evaluate the predictive performance of the two calibration equations.

The results of this study show that the inverse equation has excellent predictive performance for the calibration equation of the capacitive humidity sensor. The classical equation has better accuracy, and the inverse equation has better precision for the predictive performance of resistive humidity sensors. If the instrument response has good repeatability, the inverse equation performs excellently for the nine data sets collected in the literature. If the repeatability of the instrument response is poor, two calibration equations have similar predictive performance. The CV value of measurement in standard conditions is used as a criterion. A CV value of less than 5% can be used as a threshold basis for the inverse calibration equation.

## Figures and Tables

**Table 1:** Published data in the literature for evaluating the predictive performance of two calibration equations.

Study	Equipment	Target	Standard Range	Response Range	Calibration Equation	Statistic Criteria
Mulholland and Hibbert [21]	HPLC ^1^	Daidzein	0.162–10.96 mg/50 mL	0.243–30.75 peak area	Linear y = X^1.1^	R^2^, residual plot
Desimoni [22]	Flow injection analysis	Sulfides	0.88–81.2 μm	0.170–15.94 μA	Linear	R^2^, residual plot
Lavagnini and Magno [23]	GC-MS ^2^	Chloromethane	0~4 μg/L	0.111975~0.465813 peak area ratio	Linear polynomial	s, residual plot
Ortiz et al. [24]	Pulse polarography	Benzaldehyde	0.0198~0.1740 mnol/L	0.033~0.366 μA	Linear	Residual plots, S
Rawski et al. [25]	Spectrophotometry	Albumin	0~20 μg/mL	0~450 peak height × 10^−3^	Linear	Lack of fit, R^2^
Desharnais et al. [26]	LC-MS ^3^	Cocaine	5~1000 ng/mL	0.049~9.209 area ratio	Linear	Partial F-test
Martin et al. [27]	HPLC	Blood	0~90 ng/mL	0.002~0.272 area ratio	High-order polynomial	R^2^ Residual plots
Martin et al. [28]	LC-QqQ-MS ^4^	PrP		2150–3,054,469	Linear	R^2^
	array					
Lavin et al. [29]	BICELLS ^5^	Anti-IgG	1~100 μg/mL	0.00~6.14	Polynomial	AICs ^5^, R^2^

Note: 1. HPLC: high-performance liquid chromatography; 2. GC-MS: gas chromatography/mass spectrometry; 3. LC-MS: liquid chromatography–tandem mass spectrometry; 4. LC-QqQ-MS: liquid chromatography–mass spectrometry; 5. BICELLS: biophotonic sensing cells.

**Table 2:** The specifications of two humidity sensors.

	Resistive Sensor	Capacitive Sensor
Name	THT-B121	HMP 140A
Sensing element	Macro-molecule HPR-MQ	HUMICAP
Operating range	0–60 °C	0–50 °C
Measuring range	10–99% RH	0–100%
Nonlinearity and repeatability	±0.25% RH	±0.2% RH

**Table 3:** The predictive performance of two calibration equations for a capacitive humidity sensor.

Criterion	Classical Equation	Inverse Equation
e_i,min_	−1.9395	−1.0464
e_i,max_	0.2198	0.2414
MAE	0.9855	0.4944
RMSE	1.229	0.6064

**Table 4:** The predictive performance of two calibration equations for the resistive humidity sensor.

Criterion	Classical Equation	Inverse Equation
e_i,min_	−1.9150	−0.9950
e_i,max_	0.7311	0.8908
MAE	0.5431	0.5536
RMSE	0.4894	0.4807

**Table 5:** The criteria of the calibration regression equations for the chloromethane concentration (x_i_) and the ratio peak area (y_i_) for GC-MC.

Classical Equation	$R^{2}$	Residual Plots
1. y=0.0187+0.089x	0.9842	F.P.
2. $y=0.0113+0.1158x-0.0071x^{2}$	0.9893	U.D.
3. y=0.3033(1−exp⁡(−0.2245x))	0.9863	U.D.
4. y=0.0109+0.7477(1−exp⁡(−0.1585x))	0.9873	U.D.
5. $y=0.1232x^{0.7874}$	0.9867	F.P.
**Inverse Equation**	$R^{2}$	**Residual Plots**
1.x=−0.1782+10.7494 y	0.9773	F.D.
2. x = $-0.1114+8.920y+4.9491y^{2}$	0.9791	U.D.
3. x =1911.776(1−exp⁡(−0.0053y))	0.9691	U.D.
4. x=−0.1899+925.325(1−exp⁡(−0.012y))	0.9841	U.D.
5. x $=12.9265y^{1.2093}$	0.9793	F.P.

Note: F.P.: fixed pattern; U.D.: uniform distribution.

**Table 6:** The criteria for evaluating the predictive ability of these calibration equations for measuring chloromethane concentration with GC-MS.

Criterion	Classical Equation	Inverse Equation
e_i,min_	−0.3859	−0.3835
e_i,max_	1.4983	0.9350
MAE	0.4758	0.3328
RMSE	0.2695	0.2043

**Table 7:** The calibration regression equation and criteria for the albumin concentrations and the peak height with spectrophotometry.

Classical Equation	$R^{2}$	s_y_	Residual Plots
1. y−43.5066+22.1861	0.9651	29.081	F.P.
2. $y=0.9519+36.726x-0.7250x^{2}$	0.9980	5.5069	F.P.
3. y=626.5993(1−exp⁡(−0.0635x))	0.9985	6.0221	U.D.
4. y=−1.9587+623.7546(1−exp⁡(−0.0646x))	0.9985	6.1770	U.D.
5.$\;y=57.5278x^{0.6959}$	0.9939	12.184	F.P.
**Inverse Equation**	$R^{2}$	**s_x_**	**Residual Plots**
1. x=−1.5344+0.0435y	0.9651	1.2876	F.P.
2. $x=0.2443+0.0164y+5.8629\times 10^{-5}y^{2}$	0.994	0.5430	U.D.
3. $\;x=9363.3Exp(1-4.18\times 10^{-6}y)$	0.9522	1.5030	F.P.
4. $\;x=-1.5372+6695.5366(1-\exp(-6.2466\times 10^{-6}y))$	0.9646	1.3361	F.P.
5. $x=0.0023y^{1.4809}$	0.992	0.6153	U.D.

**Table 8:** The criteria for evaluating the predictive ability of these calibration equations for measuring the albumin concentrations using spectrophotometry.

Criterion	Classical Equation	Inverse Equation
e_i,min_	−0.4849	−0.7791
e_i,max_	1.1608	1.3347
MAE	0.4770	0.5416
RMSE	0.4147	0.4214

**Table 9:** The criteria for evaluating the predictive ability of these calibration equations for the measurement of anti-IgG by biophotonic sensing cells.

Criterion	Classical Equation	Inverse Equation
e_i,min_	−8.0672	−10.2422
e_i max_	4.3461	5.8671
MAE	2.2862	2.2715
RMSE	2.9587	3.3921

**Table 10:** The criteria for evaluating the predictive ability of these calibration equations for measuring drug concentration in blood with an HPLC assay.

Criterion	Classical Equation	Inverse Equation
e_i,min_	−0.5011	−0.475
e_i,max_	0.4713	0.482
MAE	0.1924	0.1847
RMSE	0.1171	0.1110

**Table 11:** The criteria for evaluating the predictive ability of these calibration equations for measuring the EtP compound by QqQ-MS.

Criterion	Classical Equation	Inverse Equation
e_i,min_	−33.0861	−34.4825
e_i,max_	22.0372	23.6656
MSE	15.6931	15.5105
RMSE	12.6058	13.3721

**Table 12:** The criteria for the evaluation of the predictive ability of these calibration equations for the measurement of sulfides by flow injection analysis.

Criterion	Classical Equation	Inverse Equation
e_i,min_	−0.3948	−0.1498
e_i,max_	0.4439	0.2781
MSE	0.2134	0.1355
RMSE	0.2449	0.1137

**Table 13:** The criteria for evaluating the predictive ability of these calibration equations for the measurement of daidzein with HPLC analysis.

Criterion	Classical Equation	Inverse Equation
e_i,min_	−0.0698	−0.1562
e_i,max_	0.1701	0.0684
MAE	0.0837	0.0823
RMSE	0.0668	0.073

**Table 14:** The criteria for evaluating the predictive ability of these calibration equations for measuring cocaine concentration by LC-MS-MS.

Criterion	Classical Equation	Inverse Equation
e_i,min_	−71.2154	−82.0002
e_i,max_	46.0926	36.4611
MAE	47.4945	27.3709
RMSE	35.4701	12.6038

**Table 15:** The criteria for evaluating the predictive ability of these calibration equations for measuring benzaldehyde using pulse polarography.

Criterion	Classical Equation	Inverse Equation
e_i,min_	−0.02174	−0.01608
e_i,max_	0.00858	0.007406
MAE	0.008197	0.006503
RMSE	0.009711	0.007014

**Table 16:** Comparison of the predictive performance of two calibration equations from this study and the literature data.

	RE_MAE_^1^ (Accuracy)	RE_RMSE_^2^ (Precision)
I. Hygrometer		
1. Capacitive	49.83%	50.66%
2. Resistance	−1.93%	1.78%
II. Literature data 1. GC-MS [23] 2. Flow injection Analysis [22] 3. LC-MS-MS [26] 4. Pulse polarography [24] 5. Spectrophotometry [25] 6. BICELLS (biophotonic sensing cells) [29] 7. HPLC [27] (drug in blood) 8. LC-QqQ-MS [28] 9. HPLC [21] (daidzein)	48.56% 36.5% 42.37% 20.67% −13.54% −0.64% 4.0% 1.16% 1.67%	46.53% 53.57% 64.47% 27.78% −1.62% 14.65% 5.2% −6.08% −9.28%

Note: 1. RE_MAE_ = (MAE_cla_ − MAE_inv_)/MAE_cla_, where MAEcla is the MAE value of the classical equation, and MAEinv is the MAE value of the inverse equation. The RE_MAE_ is used to evaluate the accuracy. 2. RE_RMSE_ = (RMSE_cla_ − RMSE_inv_)/RMSE_cla_, where RMSE_cla_ is the RMSE value of the classical equation, and the RMSE_inv_ is the RMSE value of the inverse equation. The RE_MAE_ is used to evaluate the precision.

## Data Availability

Data is unavailable due to a statement is still required.
